# Multifunctional Biological Performance of Electrospun PCL Scaffolds Formulated with Silver Sulfide Nanoparticles

**DOI:** 10.3390/polym17020230

**Published:** 2025-01-17

**Authors:** María del Carmen Torres-Pedroza, Ariadna Fernanda Martínez-Ávila, Karla Juarez-Moreno, Miriam Estevez, Lorena Álvarez-Contreras, Martha Elena Cruz-Soto, Lucero Granados-López, Noé Arjona, Beatriz Liliana España-Sánchez

**Affiliations:** 1 Centro de Investigación y Desarrollo Tecnológico en Electroquímica SC, Parque Tecnológico Querétaro s/n Sanfandila, Pedro Escobedo, Querétaro 76703, Mexico; mtorres@cideteq.mx (M.d.C.T.-P.); wvelazquez@cideteq.mx (N.A.); 2 Facultad de Ciencias Naturales, Universidad Autónoma de Querétaro, Av. De las Ciencias s/n Juriquilla, Querétaro 76230, Mexico; amartinez174@alumnos.uaq.mx; 3 Centro de Física Aplicada y Tecnología Avanzada, Universidad Nacional Autónoma de México, Blvd. Juriquilla No. 3001, Querétaro 76230, Mexico; kjuarez@fata.unam.mx (K.J.-M.); miries@fata.unam.mx (M.E.); 4 Centro de Investigación en Materiales Avanzados S.C. Complejo Industrial Chihuahua, Chihuahua 31136, Mexico; lorena.alvarez@cimav.edu.mx; 5 Coordinación Nacional de Investigación en Ciencias de la Salud, Universidad del Valle de México Campus Querétaro, Blvd. Juriquilla 1000 A, Del. Santa Rosa Jáuregui, Querétaro 76230, Mexico; martha.cruzso@uvmnet.edu; 6 Anatomía Patológica Querétaro, Hospital La Joya, Torre Momentum, Paseo de la República No. 13020, Querétaro 76100, Mexico; dralucerogranados@gmail.com

**Keywords:** electrospun scaffolds, PCL, antibacterial, biocompatibility, tissue regeneration, silver sulfide nanoparticles

## Abstract

Our work describes the green synthesis of silver sulfide nanoparticles (Ag_2_S NPs) and their formulation into polycaprolactone fibers (PCL), aiming to improve the multifunctional biological performance of PCL membranes as scaffolds. For this purpose, an extract of rosemary (*Salvia rosmarinus*) was employed as a reducing agent for the Ag_2_S NPs, obtaining irregular NPs and clusters of 5–60 nm, with a characteristic SPR absorption at 369 nm. Ag_2_S was successfully incorporated into PCL fibers by electrospinning using heparin (HEP) as a stabilizer/biocompatibility agent, obtaining nanostructured fibers with a ca. 500–800 nm diameter. Different amounts of Ag_2_S NPs (0.05, 0.5, and 1 wt.%) enhanced the nanostructured membranes’ surface polarity and mechanical performance, with a controlled ion release after 6 days submerged in PBS solution, determined by cyclic voltammetry. As a result, PCL/HEP/Ag_2_S scaffolds exhibit high antibacterial performance (80–90%) at early stages of contact (3 h) against *E. coli* and *S. aureus*. Also, cytotoxicity analysis demonstrated that the nanostructured membranes are biocompatible and exhibit high fibroblast cell regeneration, which is optimal for their application as scaffolds. To validate the regenerative response of PCL/HEP/Ag_2_S scaffolds, controlled wounds were induced in Wistar rats, presenting a favorable healing response by contact with PCL/HEP/Ag_2_S 1%, compared with the untreated wound. Our results indicated that nanostructured scaffolds enable the development of novel nanomaterials with multifunctional biological performance.

## 1. Introduction

The use of nanomaterials as an innovative technology in developing materials for tissue engineering has set the standard for designing composite systems that are optimal for replacing damaged tissues. Scaffolds represent a functional construct platform for successful tissue regeneration, characterized as non-toxic and biodegradable hosts that promote the growth, differentiation, and proliferation of cells on the surface [1]. However, common complications associated with scaffold rejection in the human body include bacterial surface contamination/infection [2] and poor biocompatibility [3], translating into the failure of treatments and prolonged tissue regeneration response. Currently, the use of antimicrobials in interconnected polymer networks represents a viable alternative in the successful fabrication of scaffolds, allowing the production of biocompatible substrates to improve tissue growth, combined with bactericidal performance [4,5]. The specific requirements of nanostructured scaffolds formulated with non-toxic antimicrobials include a high degree of dispersion into the polymer matrix to improve the surface reactive area, mimic the extracellular matrix (ECM) of tissues, and improve mechanical performance, biocompatibility, and biodegradability [6,7].

Polymer nanofibers have demonstrated high efficiency in developing nanostructured scaffolds due to their easy fabrication and standardization with diverse polymer matrices [8,9], including polyvinyl alcohol [10], chitosan [11], polycaprolactone [12], collagen [13], and gelatin [14], demonstrating high biocompatibility, drug delivery capacity, and the ability to design advanced scaffolds for regenerative medicine. In this regard, nanofibers can be fabricated with several techniques, including self-assembly, 3D bioprinting, template synthesis, melt blowing, melt spinning, and electrospinning. Despite electrospinning technology allowing the fabrication of well-controlled fiber morphology (50 nm to 500 µm), the NPs’ dispersion during their formulation into the dissolved polymer plays a critical factor in the successful performance of nanostructured polymer nanofibers. According to our previous work [15], electrospun polymer nanocomposites within situ-synthesized silver NPs (AgNPs) allowed the production of chitosan/AgNPs fibers of ca. 80–220 nm, with embedded spherical NPs of ca. 8 nm. Also, Hernández-Orozco et al. [16] demonstrated the fabrication of polyethersulfone (PES) with different amounts of commercial AgNPs to obtain nanostructured fibers of ca. 400–800 nm. In both cases, the intrinsic antibacterial features of AgNPs were successfully transferred into the fibers, demonstrating a high bactericidal response against pathogens such as *E. coli*, *S. aureus*, and *P. aeruginosa*, attributed to the controlled release of Ag^+^ species supported in the nanofibers. However, little is known about the use of NPs capable of controlling their Ag^+^ release and their impact on combined bactericidal/biocompatibility performance during the formulation of electrospun scaffolds.

Silver sulfide nanoparticles (Ag_2_S NPs) have gained attention due to their notable bactericidal performance [17,18,19]. This phenomenon is attributed to the silver–sulfur interaction by sulfidation, which controls the Ag^+^ release and reduces their toxicity. It also synergizes the bactericidal response through membrane damage, producing irreversible bacterial disruption and death [20]. Our work aims to improve the combined bactericidal/biocompatibility performance of electrospun PCL with green-synthesized Ag_2_S NPs, including the NPs’ characterization, the physicochemical analyses of fibers, and the Ag^+^ release of membranes by an electrochemical method. Also, we determined the bactericidal response of PCL/Ag_2_S nanocomposites against *S. aureus* and *E. coli*. Finally, we evaluated the in vivo tissue regeneration response in controlled wounds, obtaining multifunctional nanocomposites with potential applications as wound dressing material.

## 2. Materials and Methods

Silver nitrate (AgNO_3_, ACS reagent, 99.0% purity) was purchased from Sigma Aldrich (Saint Louis, MO, USA). Sodium sulfide (Na_2_S nonahydrate, 98.0% purity) was purchased from Karal S.A. de CV (Leon, Mexico). Rosemary leaves (*Salvia rosmarinus*) were purchased at the municipal market (Querétaro, Mexico). Polycaprolactone (PCL, Mw. 80,000 g/mol), heparin, methanol, and chloroform were purchased from Sigma Aldrich for electrospun membrane fabrication.

### 2.1. Green Synthesis of Ag_2_S Nanoparticles

The green synthesis of Ag_2_S NPs was performed according to A. M. Awwad et al. [19], with slight modifications. Briefly, 10 g of rosemary leaves was washed and cut. Leaves were mixed with 200 mL of deionized water and boiled at 96 °C for 20 min. The solution was filtered and stored at 4 °C. For the Ag_2_S synthesis, 10 mL of rosemary extract was mixed with 0.1 g AgNO_3_ and Triton X100 (1 wt.%) and sonicated at 20 kHz and 60% amplitude for 2 min. An amount of 10 mL of sodium sulfide was dripped, and then the final solution sonicated for 2 min, obtaining a characteristic gray-color solution of Ag_2_S NPs. After the ultrasound reaction, the mix was centrifuged thrice (600 rpm/10 min) and washed with water/ethanol. Ag_2_S was dried at 60 °C for 1 h.

### 2.2. Electrospun Scaffold Formulation of PCL/Ag_2_S Nanocomposites

PCL pellets (10 wt.%) were dissolved in a chloroform/methanol 80/20 mixture at room temperature. Different amounts of Ag_2_S NPs (0.05, 0.5, and 5 wt.%) were incorporated by sonication (2 min/20 kHz). To improve the biocompatibility of the membranes, 5 wt.% heparin was added and stirred for 1 h. The polymer suspension was electrospun using a GIGA IE^®^ (Querétaro, México) rotatory electrospinning at 15 kV, with a flow rate of 6 µL/h and 15 cm distance, for 6 h at 40% humidity. Previous parameter adjustments were performed to establish the final electrospinning conditions (see Table S1 in the Supplementary Materials).

### 2.3. Structural Characterization of Ag_2_S NPs and PCL/HEP/Ag_2_S Membranes

UV–Vis spectroscopy was employed to determine the surface plasmon band of Ag_2_S NPs using a BIOBASE BK-UV1800 PC (Jinan, China) from 300 to 800 nm. Raman spectroscopy was performed using a micro-Raman HORIBA XploRA (Jobin Yvon, Kyoto, Japan) coupled with a CCD detector with a laser λ = 785 nm. The morphology and chemical composition of Ag_2_S NPs were determined by High-Resolution Transmission Electron Microscopy (HRTEM) using a Jeol JEM 220 FS + CS (Akishima, Japan) equipped with a Tungsten filament operated at 200 kV. The crystal structure was evaluated by X-ray diffraction (XRD) using a Bruker D8 Advance (Billerica, MA, USA) coupled with a Cu Kα λ = 1.541 Å, of 10–90 in 2θ.

Scanning Electron Microscopy (SEM) micrographs of the PCL/HEP/Ag2S membranes were obtained using a JSM-7401F microscope (Jeol, Akishima, Japan) at 2 kV. The mechanical properties of the dried electrospun scaffolds (elasticity, strain, and elongation %) were measured in a Zwick/Roell tensile testing machine model Z005 (Ulm, Germany) (at 30% humidity, 22.1 °C), according to ASTM D-3822 [21]. The tensile loads of each sample were adjusted through crosshead displacement, connected to a 500 N load cell. The tensile assay was performed at 10 mm/min, using thin-film clamps. Contact angle (CA) measurements were determined with a goniometer KRAUSS DSA30 (Hamburg, Germany), and a water drop was placed on the surface of each sample for 30 s. The Ag release in the electrospun PCL/HEP/Ag_2_S membranes (4 cm^2^) submerged in phosphate-buffer solution (PBS, 0.1 M pH 7.2) was calculated by cyclic voltammetry [16] using a Gamry Reference 3000 (Warminster, PA, USA), coupled with a three-electrode system with Ag/AgCl as an electrode reference, a graphite bar as the contra electrode, and a glassy carbon electrode coated with 10 µL ink of carbon Vulcan/Nafion (see Figure S1a in the Supplementary Materials). The anodic stripping voltage (ASV) test was applied with a reducing potential (−1 V vs. Ag/AgCl) for 600 s at 1500 rpm. A linear sweep voltammogram from 0.5 to 0.7 V vs. Ag/AgCl was obtained using 50 mV/s as the speed for 1 cycle. Measurements were performed every 24 h for 9 days, calculating the Ag concentration (µg/mL) released over time according to the AgNO_3_ calibration curve (see Figure S1b in the Supplementary Materials).

### 2.4. Antibacterial Activity

The antibacterial activity (AA) of Ag_2_S NPs (625–5000 µg/mL suspended in PBS) was assessed using the microdilution method, using strains of Gram (−) *Escherichia coli* (ATCC #25922) and Gram (+) *Staphylococcus aureus* (ATCC #6538). Both microorganisms were grown in Mueller Hinton broth (MH). An optical density of 600 nm was used to adjust an inoculum cultured for 16 h to 1 × 10^5^ Colony Forming Units (CFU)/mL. A 1:1 NP/bacterial suspension mix was then shaken and incubated at 37 °C for 3 and 24 h. After NP/bacteria interaction, an aliquot (50 µL) was plated in MH agar. The AA percentage was calculated using our previously reported method [22,23].

For the PCL/HEP/Ag_2_S membranes, 1 cm^2^ squares were cut and sterilized for 20 min with a UV lamp. A humidity chamber system was employed for the bacteria/membrane interaction [16,22]. In each surface membrane, 20 µL of bacterial suspension (1 × 10^5^ CFU/mL) was plated and incubated for 3 and 24 h. After the interaction, samples were washed with 3 mL of sterile phosphate buffer (PBS), and an aliquot (50 µL) was plated in MH agar. All the evaluations were performed in three independent duplicated experiments.

### 2.5. Biocompatibility Analysis of Electrospun PCL/Ag_2_S Nanocomposites

The cytotoxicity of the PCL/HEP/Ag_2_S membranes in contact with rat 3T3-L1 fibroblasts was assessed using the resazurin reduction assay, a colorimetric method to measure active cellular metabolism. Fibroblasts were seeded into a 96-well plate (approximately 10,000 cells per well) and incubated for 24 h at 37 °C and 5% CO_2_ in 100 µL of DMEM to promote cell adhesion. Afterward, 1 cm^2^ membrane samples were added to each well to analyze cell/scaffold interactions. Cells without membranes were a positive control and cells with 10 wt.% resazurin was used as the negative control. The cells were exposed to the membranes for 24 h under the same culture conditions. Following the cell/membrane interaction, the samples and media were removed, and the cells were rinsed with PBS. Subsequently, the 3T3-L1 fibroblasts were incubated in a mixture of DMEM and 10% resazurin for 4 h at 37 °C and 5% CO_2_. Cell viability was determined by measuring resazurin reduction via UV–Vis absorbance at 570/600 nm (Thermo Scientific MultiScanGo, Waltham, MA, USA). All experiments were conducted in triplicate.

### 2.6. In Vivo Healing Response of Wounds with PCL/Ag_2_S Scaffolds

In vivo, the PCL/HEP/Ag_2_S membranes were validated as wound dressing materials in controlled injuries at the dorsum of Wistar rats, in agreement with the bioethical protocol CBUVM22024. For this purpose, male Wistar rats (ca. 250 g) were anesthetized and their dorsum shaved, producing an incision of 1 cm in length in the back. Squares of 2 cm were adhered to the injury and changed every 24 h for 14 days, including a model without a membrane as an untreated control model. Photographs of the healing evolution were taken, and after 14 days, the animals were sacrificed. Lesioned skin tissue was stained with hematoxylin/eosin (H&E) and analyzed by histopathology [5].

Each biological analysis of the developed scaffolds was conducted with a minimum of three independent experiments. The data obtained are presented as the mean value ± standard deviation (SD). Statistical analyses included one-way ANOVA, evaluated using Tukey’s test. A multiple comparison test was performed to determine differences between groups. A significance level of *p* < 0.05 was considered statistically significant for the antibacterial activity of the scaffolds against *S. aureus* and *E. coli*.

## 3. Results

### 3.1. Synthesis of Ag_2_S Nanoparticles

**Figure 1:**
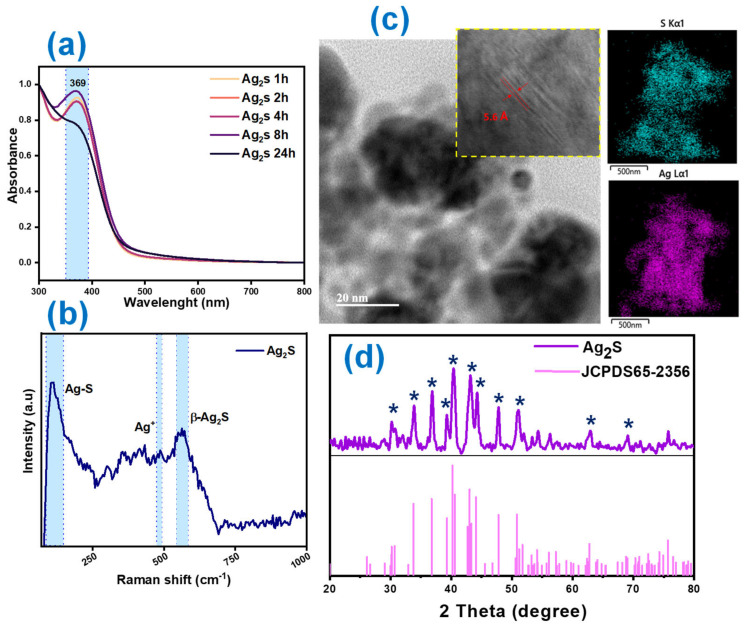
Structural characterization of Ag_2_S NPs synthesized from ultrasound-assisted green synthesis with *Salvia rosmarinus* extract. (**a**) UV–VIS spectra of Ag_2_S and its stability suspension, (**b**) RAMAN spectra, (**c**) HRTEM micrograph of Ag_2_S, including the crystal lattice and the sulfur (S) and silver (Ag) mapping, and (**d**) XRD pattern of Ag_2_S and its comparison with the JCPDS 65-2356 card. * Indicate the position of the principal planes characteristic of the acanthite structure of Ag_2_S NPs.

The green synthesis of antimicrobial nanomaterials has benefitted the fabrication of NPs that can eradicate pathogen growth, avoiding the use of toxic reducing agents that represent health risks [24]. The green synthesis and the characterization of Ag_2_S NPs using rosemary extract (*Salvia rosmarinus*) were performed, according to the report by Awwad—et al. [19], as presented in Figure 1. The UV–Vis spectra (Figure 1a) of Ag_2_S present a characteristic surface plasmon resonance peak (SPR) at 369 nm for Ag_2_S NPs [25], suggesting that the chemical composition of rosemary extract (polyphenols, flavonoids, and terpenes) intervened in the reduction of AgNO_3_ and Na_2_S through the catalytic reaction induced by the ultrasound waves. Figure 1b shows the Raman spectra of Ag_2_S, demonstrating the Ag-S vibration at 101 cm^−1^, the Ag^+^ peak at 490 cm^−1^, and the reversible photoluminescence attributed to the longitudinal symmetric modes of the β-Ag_2_S vibrations [26,27]. Additional multiple peaks in the Raman spectra indicate the photothermal degradation of Ag_2_S NPs by the laser excitation applied. HRTEM micrographs (Figure 1c) describe the successful synthesis of semispherical and agglomerated Ag_2_S NPs coated with an organic layer associated with the traces of rosemary extract, with an average diameter of 5–60 nm. Also, a well-defined crystal lattice is observed, with an interplanar distance of 5.6 Å. The chemical composition reveals the presence of Ag and S species, with ca. 63.7% Ag vs. 36.3% S. A detailed crystalline structure was obtained by XRD patterns, indicating the formation of a monoclinic structure with an acanthite crystal phase of Ag_2_S NPs, with the presence of peaks located at 30.02, 33.9, 36.9, 44.3, and 62.6 at 2θ, which correspond to the planes [−112], [−121], [111], [200], and [220], respectively [28], compared with the JCPDS 65-2356 card of monoclinic Ag_2_S nanostructures [29].

The AA of Ag_2_S NPs was evaluated against *E. coli* and *S. aureus* (see Figure S2 in the Supplementary Materials). Despite the differences in the bactericidal behavior between both microorganisms associated with their specific bacterial wall composition, at the early stage of bacteria/NP interaction (3 h), the bactericidal response of 1250 µg/mL of Ag_2_S NPs is ca. 89%; complete bacterial inhibition (99.99%) is performed after 24 h against *S. aureus*, indicating that their intrinsic bactericidal activity can be successfully transferred into the PCL matrix.

### 3.2. Morphology and Physicochemical Properties of Electrospun PCL/Ag_2_S Scaffolds

**Figure 2:**
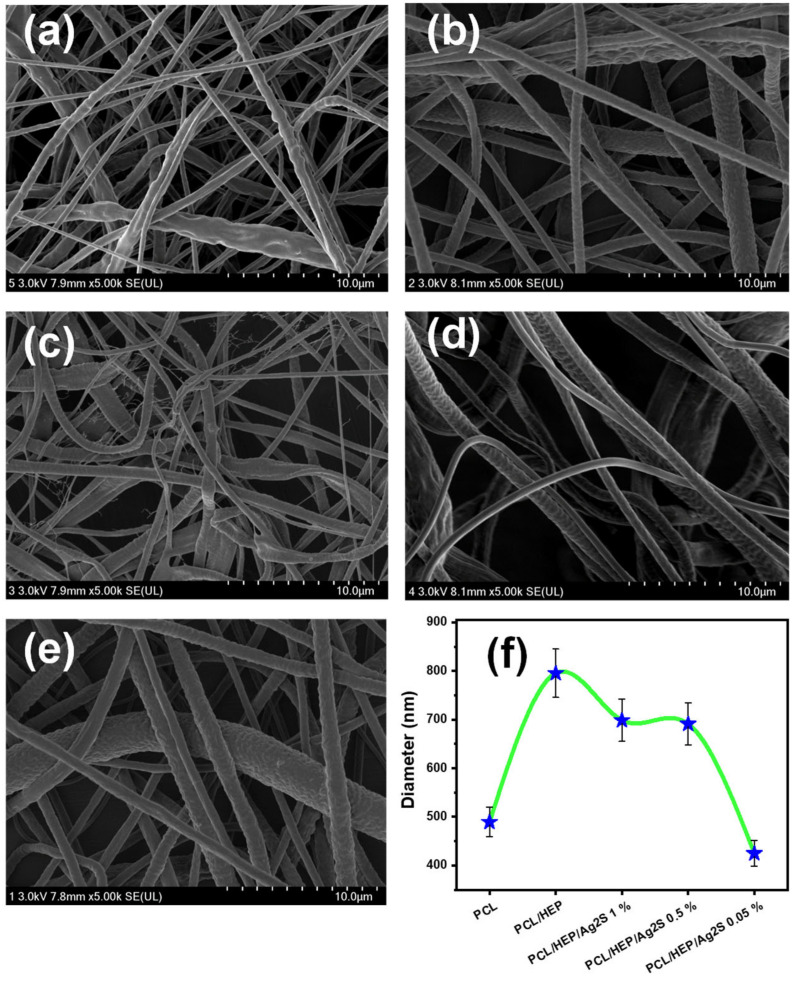
SEM images of electrospun PCL/Ag_2_S scaffolds. (**a**) PCL, (**b**) PCL/HEP, (**c**) PCL/HEP/Ag_2_S 0.05%, (**d**) PCL/HEP/Ag_2_S 0.5%, (**e**) PCL/HEP/Ag_2_S 1%, and (**f**) comparison of average diameter size of fibers. Stars represent the mean of diameter size of electrospun nanofibers of each sample.

Figure 2 shows the representative SEM micrographs of pristine PCL and the incorporation of HEP and Ag_2_S NPs. The morphology of the electrospun PCL fibers (Figure 2a) indicates the presence of irregular and rough fibers with pores, with a diameter of 400–1800 nm, in agreement with the report by Can-Herrera et al. [30] at 15 kV. An improvement in the surface roughness and the decrease in pore defects is observed by adding HEP (Figure 2b), with a diameter of ca. 800 nm. It has been claimed that HEP is a negatively charged natural polysaccharide used as a compatibility agent in PCL fibers, able to perform tissue regeneration by its intrinsic anti-proliferative effect [31]. Despite the HEP increase in the fiber mat size, the fiber homogeneity was improved. In addition, the incorporation of Ag_2_S NPs (Figure 2c–e) slightly decreases the diameter of fibers associated with the NPs’ dispersion (Figure 2f) and the intrinsic conductivity of NPs in the polymer solution, promoting the formation of irregular mats. The presence of nanostructures within the electrospun PCL interferes with the Taylor cone formation due to the increased Coulomb forces needed to perform the fiber formation [32]. The histograms are included in Figure S3, Supplementary Materials.

**Figure 3:**
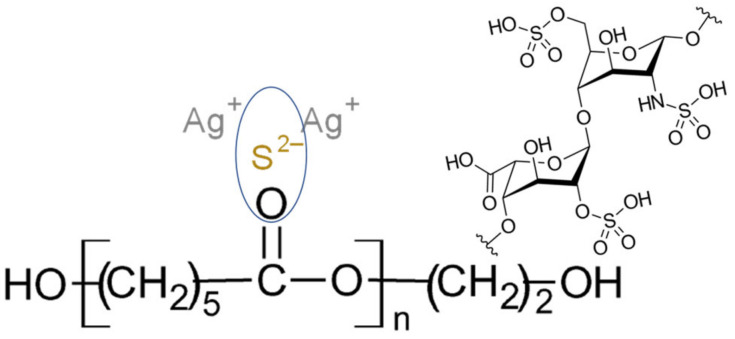
Schematic representation of the molecular interaction of the PCL polymer solution with HEP and Ag^+^ ions of the Ag_2_S NPs during the electrospinning process.

**Figure 4:**
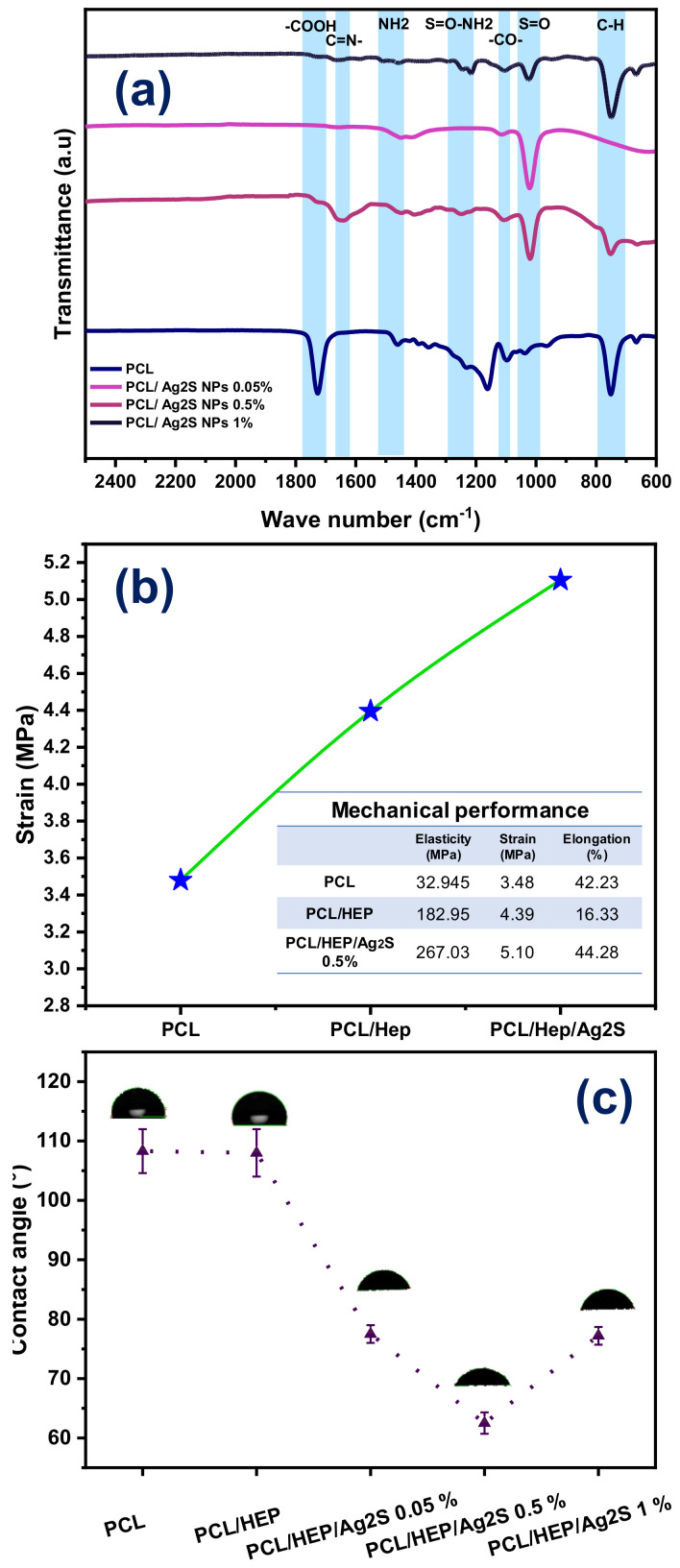
Physicochemical properties of electrospun PCL/Ag_2_S scaffolds. (**a**) Chemical composition by FTIR spectra, (**b**) mechanical performance, and (**c**) contact angle measurements.

By incorporating HEP into the electrospun fibers, the fiber diameter was increased. The above is attributed to the covalent bonding of HEP with the functional groups of PCL (Figure 3), which increases the density and apparent molecular weight while simultaneously raising the viscosity of the polymer solution, an essential parameter for a successful electrospinning process [33]. Also, due to the high affinity of silver ions for electronegative atoms such as oxygen, Ag_2_S NPs could coordinate with the carbonyl groups of the PCL structure. This coordination leads to the S=O bond observed on the FTIR spectra (Figure 4a), potentially altering the surface tension of the solution, the fiber formation dynamics, and the intrinsic conductivity upon the addition of NPs into the polymer solution. The above results in the formation of irregular fibers and interferes with the formation of the Taylor cone due to the increased Coulomb forces, facilitating the fibers’ formation [33]. Consequently, the attraction between the droplet and the collector is higher, resulting in the formation of thinner fibers.

The chemical composition of green-synthesized Ag_2_S NPs and their impact on the formulation of nanostructured PCL/Ag_2_S scaffolds was determined by FTIR spectroscopy (Figure 4a). The characteristic peaks of PCL are situated in the 1680–1750 cm^−1^ range. However, a prominent peak is observed at 1730 cm^−1^, corresponding to the C=O bond associated with carbonyl groups and the stretching of the OH group in carboxylic acid. Additionally, symmetric C–O–C bands were identified at 1230 and 1162 cm^−1^, while the peaks in the 1470–1360 cm^−1^ range are attributed to CH_2_ stretching and OH groups [33]. With the incorporation of Ag_2_S NPs, a decrease in the intensity of the C=O and C–O– peaks was observed. This interaction suggests the disruption of hydrogen bonds due to the interaction between Ag_2_S NPs and the Van der Waals forces in the polymer, increasing surface hydrophilicity. In the PCL/HEP/Ag_2_S membranes with concentrations of 0.5% and 1%, a characteristic peak was detected at 1018 cm^−1^, corresponding to the S=O bond. This result is attributed to the bond formation between the sulfur in Ag_2_S NPs and the components of PCL and HEP.

The mechanical performance of electrospun PCL/HEP/Ag_2_S nanocomposites is critical for their successful biomedical application as scaffolds. Figure 4b shows the mechanical behavior of PCL fibers, with a strain of ca. 3 mPa and an elasticity of 32.9 mPa. It is important to note that the HEP addition enhances elasticity and the strain, with a decrease in the elongation value. However, the presence of Ag_2_S NPs (1 wt.%) benefits the mechanical performance of electrospun fibers, indicating that the well-dispersed NPs represent a reinforcement effect during the nanocomposite fabrication [34]. The stress-strain curves of PCL, PCL/HEP, and PCL/HEP/Ag_2_S 0.5% was presented in Figure S4, Supplementary Materials. Additionally, the surface polarity of nanostructured membranes was obtained (Figure 4c), where the hydrophobic behavior of pristine PCL fibers was corroborated at ca. 110° [35]. The HEP as an additive does not modify the surface polarity of the materials. However, the addition of Ag_2_S NPs decreases the surface energy of electrospun PCL/HEP/Ag_2_S nanocomposites, generating a partially hydrophilic membrane (of ca. 70°). The above suggests that the combined intrinsic chemical nature of NPs obtained by green synthesis in aqueous media with NP distribution into PCL fibers improves the hydrophilic behavior of electrospun membranes. This phenomenon is desirable for promoting cellular adhesion and biocompatibility as a suitable scaffold for cellular growth and proliferation [34]. Also, the hydrophilic behavior of the material can benefit the interaction with microorganisms suspended in an aqueous medium, where their interaction with a surface formulated with bactericidal NPs will be favored, with a satisfactory antibacterial response expected.

### 3.3. Antibacterial Performance of PCL/Ag_2_S Scaffolds

**Figure 5:**
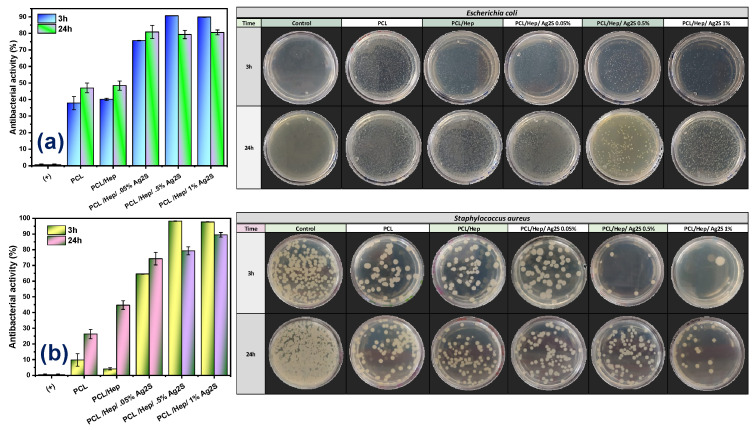
Antibacterial activity of electrospun PCL/Ag_2_S scaffolds against (**a**) *E. coli* (*p* = <0.0001), and (**b**) *S. aureus (p* = 0.0003).

The kinetics of the AA of Ag_2_S NPs against common pathogens (*E. coli* and *S. aureus*) were successfully transferred into the electrospun PCL scaffolds, as presented in Figure 5. For both microorganisms, pristine PCL fibers show a slight bactericidal response (ca. 35–40%), indicating the possible solvent traces embedded into the fibers without significant changes by adding HEP. However, the incorporation of Ag_2_S NPs is directly proportional to the bactericidal response, with an AA of ca. 80–99% against *E. coli* and an AA of ca. 75–99% against Gram (+) bacteria. This behavior is dependent on the NP amount, with higher bactericidal performance at an early stage of interaction (3 h), indicating that the enhanced surface area of the electrospun fibers and their hydrophobic behavior promote the contact between the microorganisms and the nanostructured membrane, inducing irreversible bacterial damage and death. The bactericidal response is attributed to the simultaneous generation of reactive oxygen species (ROS), DNA damage, and cell wall alteration induced by the intrinsic properties of Ag_2_S NPs [16]. Also, it has been reported that nanostructured PCL fibers allow the controlled ion release of species for bactericidal purposes [36,37], which coincides with the results obtained in this work.

### 3.4. Controlled Ion Release of PCL/Ag_2_S Scaffolds by Cyclic Voltammetry

**Figure 6:**
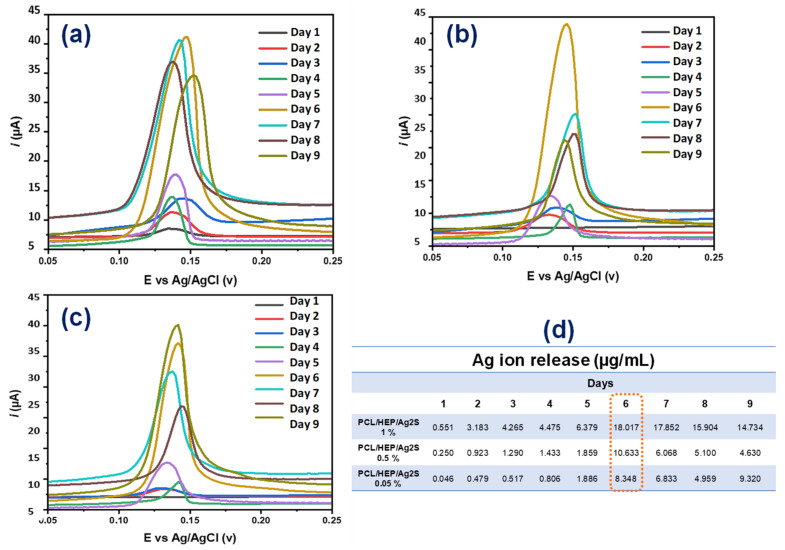
Electrochemical ion release measurements of electrospun PCL/Ag_2_S scaffolds submerged in PBS solution for 9 days under environmental conditions. (**a**) PCL/HEP/Ag_2_S 0.05%, (**b**) PCL/HEP/Ag_2_S 0.5%, (**c**) PCL/HEP/Ag_2_S 1%, and (**d**) table of Ag concentration release.

Electrochemical measurements by cyclic voltammetry were performed to determine the PCL/Ag_2_S scaffold stability in an aqueous solution and the controlled Ag^+^ release (Figure 6), considering that the anodic response of the Ag^+^ ion can be directly proportional to the concentration of species in the medium, according to our previous results [16]. Since the first day, all nanostructured membranes (Figure 6a–c) release silver species of ca. 0.045–0.5 µg/mL, which slightly increases during the next five days. After 6 days, the electrospun membrane loses its structural stability, attributed to environmental degradation through being submerged in phosphate-buffer solution 0.1 M. In this case, the nanostructured PCL/Ag_2_S scaffolds perform the controlled release of Ag^+^ species with an average concentration of ca. 8.34–18.01 µg/mL, attributed to the silver–sulfur interaction by sulfidation after 6 days, promoting the successful bactericidal response of materials.

### 3.5. Biocompatibility Assays of PCL/Ag_2_S Scaffolds

**Figure 7:**
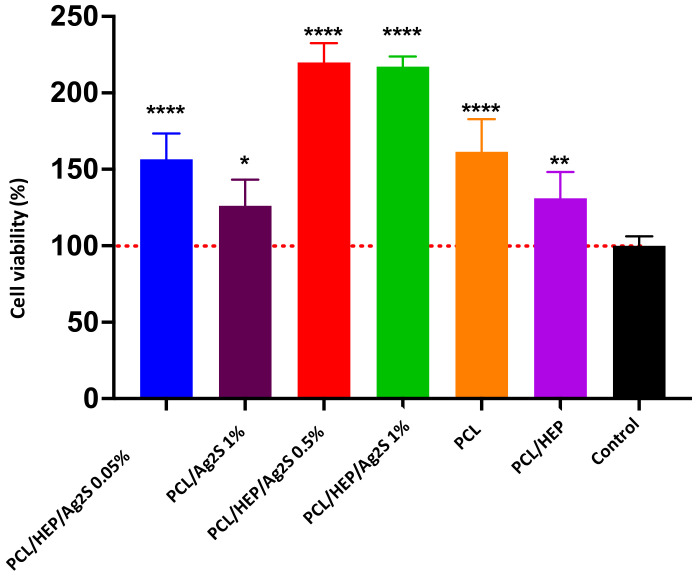
Cytotoxicity assay of fibroblasts (3T3-L1) deposited at the surface of electrospun PCL/Ag_2_S scaffolds, determined by resazurin assay. (* *p* < 0.05; ** *p* < 0.01; **** *p* < 0.0001.).

The design of electrospun PCL/Ag_2_S scaffolds significantly enhances their biocompatibility, making them suitable for applications in tissue engineering. The nanofibrous structure can provide a high surface-area-to-volume ratio, promoting cellular attachment and proliferation with improved bactericidal performance. To corroborate the biocompatibility of nanostructured membranes, 3T3-L1 fibroblast cells were used as a cytotoxicity model, as presented in Figure 7. It can be seen that the pristine PCL improves cell viability by ca. 150%, whereas the HEP does not represent significant changes in fibroblast growth. Despite the bactericidal performance of the PCL/HEP/Ag_2_S membranes, the cellular viability is increased by more than 200% compared to the control, independent of the NP amount (0.5 and 1 wt.%), demonstrating that the biosynthesized Ag_2_S NPs are biocompatible, in agreement with Suresh et al. [38]. As a result, the combination of nanostructured membranes does not generate a cytotoxic material, improving the growth of fibroblasts. This behavior can be explained in terms of the controlled release of Ag^+^ species in the Ag_2_S NPs induced by the silver–sulfur interaction, aimed at the structural conformation of electrospun membranes facilitating cell growth as a scaffold.

### 3.6. In Vivo Tissue Regeneration Response of PCL/Ag2S Scaffolds in Wistar Rat Model

The wound healing process involves partially overlapping phases: hemostasis, inflammation, proliferation, and remodeling. Fibroblasts play an essential role in each phase for efficient tissue repair. During hemostasis, fibroblasts receive chemical signals from the clot that activate and recruit them to subsequent stages. In the inflammatory phase, fibroblasts begin differentiating into myofibroblasts to contract the wound. Fibroblasts migrate to the wound site during proliferation and produce type III collagen, forming a temporary extracellular matrix. Subsequently, fibroblasts interact with endothelial cells to promote new blood vessel formation. In remodeling, fibroblasts reorganize the extracellular matrix by secreting matrix metalloproteinases (MMPs) and aligning collagen fibers. Most fibroblasts undergo apoptosis after remodeling, leaving only a small population to maintain scar tissue [39]. Fibroblasts contribute to the initial formation of granulation tissue and structural reorganization, determining scar tissue quality. They are essential cellular models for evaluating biocompatibility and the effectiveness of materials designed to promote wound repair. While keratinocytes are relevant for re-epithelialization, fibroblasts play a crucial role in the early and middle phases of repair by structuring mechanical support for new tissue. Evaluating fibroblasts helps to understand how scaffolds support this critical phase of the process [40].

**Figure 8:**
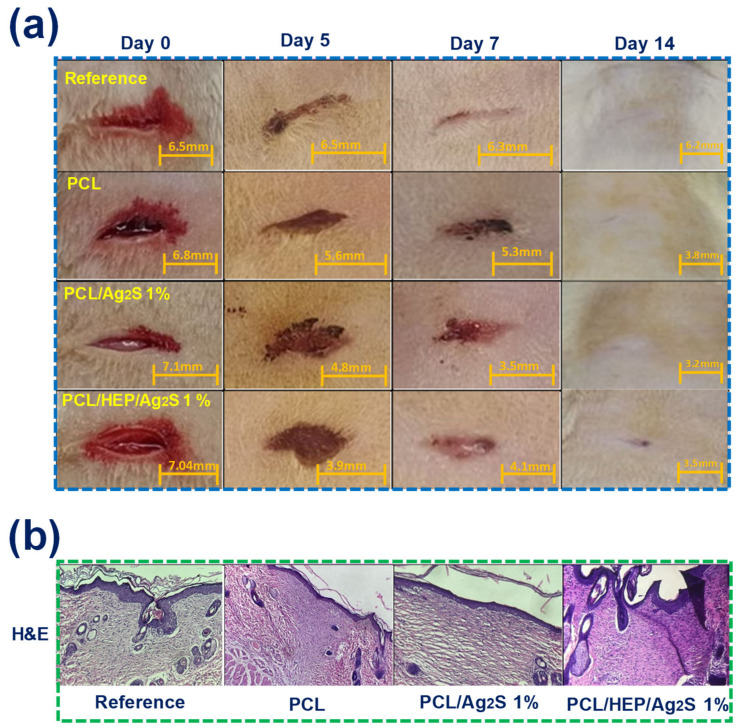
In vivo tissue regeneration response of electrospun PCL/Ag_2_S scaffolds. (**a**) Photographs of the healing response of injuries after 0, 5, 7, and 14 days of treatment. (**b**) H&E stains of injury sections.

The combined bactericidal and biocompatible effect of electrospun PCL/Ag_2_S scaffolds indicates that our nanostructured membranes can be successfully applied as a wound dressing material. Figure 8 shows the in vivo tissue regeneration response in a controlled injury produced at the dorsum of Wistar rats (bioethical protocol number CBUVM22024). Figure 8a shows the photographs of evolutive healing after 0, 5, 7, and 14 days, with wound dressing changes every 24 h. The untreated injury reveals complete healing after 14 days, with apparent evidence of scar formation. Visually, the application of nanostructured membranes represents a favorable healing evolution, with a reduced scar in the injury treated specifically with the PCL/Ag_2_S scaffold. In vivo validation results indicate significant differences in wound healing between treated and untreated injuries regarding evolution and regeneration quality. Untreated injuries showed limited recovery between day 0 and 7, with minimal wound size reduction (6.5 mm to 6.3 mm), reflecting a lower healing rate. Conversely, for wounds treated with PCL alone a significant reduction in wound size (6.8 to 3.8 mm) was observed, indicating that the use of a biodegradable barrier performs skin reparation mechanisms. In addition, the nanostructured PCL/Ag_2_S 1% scaffold exhibited a favorable healing evolution, with a significant wound size reduction (of ca. 3.9 mm). It is important to note that the HEP incorporation into the nanostructured scaffolds does not affect the wound healing mechanism, representing a balance of the inflammatory and proliferative phases that results in optimizing the tissue regeneration.

The histological evaluation of injuries after 14 days (Figure 8b) revealed significant similarities in the regenerative outcomes among the control groups and those treated with PCL-based dressings. The control group and the control PCL-treated rats exhibited laminar ortho-keratosis, small fibroblasts surrounded by abundant dense collagen, intact appendages, and a sparse presence of mast cells within the reticular dermis and adipose tissue. These findings indicate complete tissue recovery, as evidenced by the presence of hair follicles. However, a difference was noted in the control group, where an irregular detachment of keratin layers in the epidermis was observed. In contrast, the injury treated with the PCL/Ag_2_S 1% NP dressing showed a similar pattern of laminar ortho-keratosis, small fibroblasts with dense collagen deposition, intact appendages, sparse mast cells in the reticular dermis and adipose tissue, and well-formed hair follicles. These observations confirm that Ag_2_S NPs did not adversely affect tissue integrity or healing. Similarly, the tissue treated with the PCL/HEP/Ag_2_S 1% NP dressing demonstrated laminar ortho-keratosis, small fibroblasts with abundant dense collagen, intact appendages, sparse mast cells, and hair follicles. However, this tissue exhibited a more pronounced pink (eosin) coloration, suggesting an advanced regeneration stage characterized by organized collagen deposition and uniformly structured keratin in the epidermis. Considering that skin wounds often heal with scars devoid of hair follicles and glands, the repair is considered successful when it results in limited scar tissue that restores protective barrier integrity. The presence of hair follicles [39,40], as observed in tissue treated with PCL/Ag_2_S NP/HEP, further supports successful regeneration. This behavior suggests an advanced regeneration stage characterized by organized collagen deposition and uniformly structured keratin in the epidermis. These results highlight the enhanced regenerative potential of the PCL/HEP/Ag_2_S 1% NP dressing, potentially attributed to its bioactive properties that promote epidermal remodeling and collagen synthesis. These findings collectively suggest that while all treatments achieved comparable levels of tissue recovery, incorporating bioactive components such as biosynthesized Ag_2_S NPs in PCL dressings may accelerate and refine the regenerative process, emphasizing their potential for advanced wound care applications.

Wound dressings can be categorized into four primary types based on their functionality and interaction with the wound: passive, interactive, advanced, and bioactive. Passive dressings act as protective barriers, shielding the wound from physical trauma and preventing the entry of pathogens. Interactive dressings, often made from polymer-based films, enable the exchange of air and moisture while offering protection against bacterial or environmental contaminants. Advanced dressings are designed to maintain a moist wound environment, which promotes and accelerates the healing process. Lastly, bioactive dressings integrate drug-delivery systems to stimulate cellular responses in the healing process [41]. With these characteristics, the proposed dressing combines PCL (a biodegradable polymer), Ag_2_S (an antimicrobial compound), and heparin (an agent capable of modulating biological responses). This combination classifies it as both bioactive and advanced, as it integrates the antimicrobial properties of Ag_2_S NPs, cellular process-stimulating properties, and wound environment improvements provided by HEP. PCL provides a supportive matrix necessary for regeneration, combining physical barrier properties, infection control, and bioactivity potential to modulate cellular responses, which are crucial for tissue regeneration. In such dressings, the dynamic interactions between the wound and the environment enhance the healing process, overcoming the limitations of passive dressings. In chronic wounds with high exudate levels, passive dressings adhere to the wound and may cause severe pain and damage during removal.

In wound dressings for tissue regeneration, the presence of bacterial infections represents a critical challenge, as it can delay healing or hinder regeneration. Therefore, designing dressings with intrinsic antibacterial properties is essential to ensure effective recovery and prevent infectious complications in advanced clinical applications [42]. The research on antibacterial materials for wound dressings is crucial, as an excessive increase in ROS levels leads to oxidative stress, triggering strong inflammatory reactions and wound infections. Elevated ROS levels overwhelm the antioxidant capacity of cells, preventing the wound from transitioning from the inflammatory to the proliferative stage. In this context, there is a need for a more effective and comprehensive strategy to prevent bacterial colonization and infection, inhibit inflammation, and accelerate wound healing. Common antibacterial agents include metal ions, natural biological macromolecules, and photothermal agents. The use of metallic antibacterial agents is limited due to their potential toxicity [43]. Diverse studies have reported that the sulfurization of Ag NPs reduces oxidative stress, protecting biological systems from silver nanoparticle-induced toxicity. Consequently, the sulfurization of AgNPs becomes a viable strategy to mitigate the impact of biological toxicity [44]. In this study, it has been demonstrated that the sulfurization of silver nanoparticles (Ag NPs) offers a viable approach to reduce their impact on biological toxicity, which has direct implications for the development of safer and more effective materials for biomedical applications. The sulfurization of Ag NPs leads to the formation of silver sulfide nanoparticles (Ag_2_S NPs), which result in decreased ionic mobility and a controlled, sustained release of silver ions (Ag^+^) [43], as evidenced by electrochemical characterization. This release is maintained over a period of 9 days. This regulation reduces oxidative stress, protecting biological systems and promoting a more favorable environment for cellular growth, as demonstrated by the increased viability of 3T3-L1fibroblast cells. Additionally, Ag_2_S NPs generate reactive oxygen species (ROS), including hydrogen peroxide (H_2_O_2_), which contribute to their bactericidal activity by disrupting bacterial cell membranes, inhibiting essential enzymes, and causing oxidative stress. This mechanism is supported by the ability of NPs to release Ag^+^ ions upon contact with biological systems, which bind to negatively charged bacterial cell membranes, disintegrating the cell wall and exposing intracellular contents. Furthermore, Ag^+^ ions can interact with thiol groups (–SH) in bacterial enzymes, causing their inactivation and subsequent cell death [40]. As a result, the combination of these properties—ROS generation and controlled Ag^+^ ion release—results in a balanced antimicrobial activity and biocompatibility, making the incorporation of Ag_2_S NPs into PCL scaffolds essential for wound healing applications. This approach prevents infection spread while offering a promising strategy for tissue engineering and wound treatment.

## 4. Conclusions

Our work describes the integration of green-synthesized silver sulfide nanoparticles (Ag_2_S NPs) into polycaprolactone (PCL) electrospun scaffolds, demonstrating multifunctional capabilities for advanced wound healing applications, particularly bactericidal response and biocompatibility. These nanostructured membranes exhibited enhanced mechanical properties, increased surface hydrophilicity, and controlled silver ion release, contributing to their antibacterial performance against Escherichia coli and Staphylococcus aureus. Furthermore, incorporating Ag_2_S NPs improved the PCL scaffolds’ biocompatibility and regenerative capacity, as evidenced by increased fibroblast proliferation and accelerated tissue repair applied to in vivo models. This study highlights the potential of PCL/Ag_2_S nanocomposites as a promising platform for antimicrobial and tissue engineering applications, paving the way for developing innovative wound dressing materials with improved biological performance.

## Data Availability

The data presented in this study are available on request from the corresponding author due to the patent being in progress.

## Supplementary Materials

The following supporting information can be downloaded at https://www.mdpi.com/article/10.3390/polym17020230/s1: Figure S1: Ag ion release test by cyclic voltammetry: (a) three-electrode configuration and (b) calibration curve of Ag2S NPs; Figure S2: Kinetics of antibacterial activity (AA) of Ag2S NPs after 3 and 24 h of contact against Gram (−) E. coli and Gram (+) S. aureus; Figure S3: Fiber diameter distribution obtained from the electrospun PCL and PCL/HEP, and the formulation of different amounts of Ag2S NPs (PCL/HEP/Ag2S); Figure S4. Stress-strain curves of (a) PCL, (b) PCL/HEP, and (C) PCL/HEP/Ag2S 0.5%.; Table S1: Comparative table of electrospinning parameters adjustment to obtain PCL/HEP/Ag2S nanostructured scaffolds.

## References

[1] Echeverria Molina M.I., Molina M.I.E., Malollari K.G., Komvopoulos K. (2021). Design Challenges in Polymeric Scaffolds for Tissue Engineering. Front. Bioeng. Biotechnol..

[2] Serrano-Aroca Á., Cano-Vicent A., i Serra R.S., El-Tanani M., Aljabali A., Tambuwala M.M., Mishra Y.K. (2022). Scaffolds in the microbial resistant era: Fabrication, materials, properties and tissue engineering applications. Mater. Today Bio.

[3] Bakhtiari H., Nouri A., Khakbiz M., Tolouei-Rad M. (2023). Fatigue behaviour of load-bearing polymeric bone scaffolds: A review. Acta Biomater..

[4] Hernández-Rangel A., Silva-Bermudez P., España-Sánchez B., Luna-Hernández E., Almaguer-Flores A., Ibarra C., Garcia-Perez V., Velasquillo C., Luna-Bárcenas G. (2018). Fabrication and in vitro behavior of dual-function chitosan/silver nanocomposites for potential wound dressing applications. Mater. Sci. Eng. C Mater. Biol. Appl..

[5] Luna-Hernández E., Cruz-Soto M., Padilla-Vaca F., Mauricio-Sánchez R., Ramirez-Wong D., Muñoz R., Granados-López L., Ovalle-Flores L., Menchaca-Arredondo J., Hernández-Rangel A. (2017). Combined antibacterial/tissue regeneration response in thermal burns promoted by functional chitosan/silver nanocomposites. Int. J. Biol. Macromol..

[6] Martins A., Araújo J.V., Reis R.L., Neves N.M. (2007). Electrospun Nanostructured Scaffolds for Tissue Engineering Applications. Nanomedicine.

[7] Tonda-Turo C., Ruini F., Ceresa C., Gentile P., Varela P., Ferreira A.M., Fracchia L., Ciardelli G. (2018). Nanostructured scaffold with biomimetic and antibacterial properties for wound healing produced by ‘green electrospinning’. Colloids Surf. B Biointerfaces.

[8] Anjum S., Rahman F., Pandey P., Arya D.K., Alam M., Rajinikanth P.S., Ao Q. (2022). Electrospun Biomimetic Nanofibrous Scaffolds: A Promising Prospect for Bone Tissue Engineering and Regenerative Medicine. Int. J. Mol. Sci..

[9] Flores-Rojas G.G., Gómez-Lazaro B., López-Saucedo F., Vera-Graziano R., Bucio E., Mendizábal E. (2023). Electrospun Scaffolds for Tissue Engineering: A Review. Macromol.

[10] Türkoğlu G.C., Khomarloo N., Mohsenzadeh E., Gospodinova D.N., Neznakomova M., Salaün F. (2024). PVA-Based Electrospun Materials—A Promising Route to Designing Nanofiber Mats with Desired Morphological Shape—A Review. Int. J. Mol. Sci..

[11] Cui C., Sun S., Wu S., Chen S., Ma J., Zhou F. (2021). Electrospun chitosan nanofibers for wound healing application. Eng. Regen..

[12] Azari A., Golchin A., Maymand M.M., Mansouri F., Ardeshirylajimi A. (2021). Electrospun Polycaprolactone Nanofibers: Current Research and Applications in Biomedical Application. Adv. Pharm. Bull..

[13] Law J.X., Liau L.L., Saim A., Yang Y., Idrus R. (2017). Electrospun Collagen Nanofibers and Their Applications in Skin Tissue Engineering. Tissue Eng. Regen. Med..

[14] El-Seedi H.R., Said N.S., Yosri N., Hawash H.B., El-Sherif D.M., Abouzid M., Abdel-Daim M.M., Yaseen M., Omar H., Shou Q. (2023). Gelatin nanofibers: Recent insights in synthesis, bio-medical applications and limitations. Heliyon.

[15] Cortes Y.Z., Valenzuela L.M., Pena E.A.E., Sanchez B.L.E. (2021). Antibacterial Activity of Electrospun Nanocomposites Fabricated by In Situ Chitosan/Silver Nanoparticles. IEEE Trans. NanoBioscience.

[16] Hernández-Orozco M.M., Castellanos-Espinoza R., Hernández-Santos N.A., Ramírez-Montiel F.B., Álvarez-Contreras L., Arellano-Arreola V.M., Padilla-Vaca F., Arjona N., España-Sánchez B.L. (2022). Antibacterial and electrochemical evaluation of electrospun polyethersulfone/silver composites as highly persistent nanomaterials. Polym. Compos..

[17] Pang M., Hu J., Zeng H.C. (2010). Synthesis, Morphological Control, and Antibacterial Properties of Hollow/Solid Ag2S/Ag Heterodimers. J. Am. Chem. Soc..

[18] Delgado-Beleño Y., Martinez-Nuñez C., Cortez-Valadez M., Flores-López N., Flores-Acosta M. (2018). Optical properties of silver, silver sulfide and silver selenide nanoparticles and antibacterial applications. Mater. Res. Bull..

[19] Awwad A.M., Salem N.M., Aqarbeh M.M., Abdulaziz F.M. (2019). Green synthesis, characterization of silver sulfide nanoparticles and antibacterial activity evaluation. Chem. Int..

[20] Subramaniyan S.B., Megarajan S., Vijayakumar S., Mariappan M., Anbazhagan V. (2019). Evaluation of the toxicities of silver and silver sulfide nanoparticles against Gram-positive and Gram-negative bacteria. IET Nanobiotechnol..

[21] (2020). Standard Test Method for Tensile Properties of Single Textile Fibers.

[22] España-Sanchez B.L., Avila-Orta C.A., Padilla-Vaca L.F., Barriga-Castro E.D., Soriano-Corral F., Gonzalez-Morones P., Ramirez-Wong D.G., Luna-Barcenas G. (2017). Early Stages of Antibacterial Damage of Metallic Nanoparticles by TEM and STEM-HAADF. Curr. Nanosci..

[23] Salas Z.H., Ávila A.F.M., Orozco M.M.H., Peña E.A.E., Tirado L.P., Pérez L.A.B., Vaca F.P., Luna-Bárcenas G., Sánchez B.L.E. (2022). Green synthesis of copper nanoparticles and their formulation into face masks: An antibacterial study. Polym. Compos..

[24] Vanlalveni C., Lallianrawna S., Biswas A., Selvaraj M., Changmai B., Rokhum S.L. (2021). Green synthesis of silver nanoparticles using plant extracts and their antimicrobial activities: A review of recent literature. RSC Adv..

[25] Sadovnikov S.I., Kuznetsova Y.V., Rempel A.A. (2016). Ag2S silver sulfide nanoparticles and colloidal solutions: Synthesis and properties. Nano-Struct. Nano-Objects.

[26] Sadovnikov S.I., Vovkotrub E.G., Rempel A.A. (2018). Micro-Raman Spectroscopy of Nanostructured Silver Sulfide. Dokl. Phys. Chem..

[27] Alekperov O., Jahangirli Z., Paucar R. (2016). First-principles lattice dynamics and Raman scattering in ionic conductor β-Ag_2_S. Phys. Status Solidi (b).

[28] Elsaeedy H. (2019). A low temperature synthesis of Ag2S nanostructures and their structural, morphological, optical, dielectric and electrical studies: An effect of SDS surfactant concentration. Mater. Sci. Semicond. Process..

[29] Fakhri A., Pourmand M., Khakpour R., Behrouz S. (2015). Structural, optical, photoluminescence and antibacterial properties of copper-doped silver sulfide nanoparticles. J. Photochem. Photobiol. B Biol..

[30] Can-Herrera L., Oliva A., Dzul-Cervantes M., Pacheco-Salazar O., Cervantes-Uc J. (2021). Morphological and Mechanical Properties of Electrospun Polycaprolactone Scaffolds: Effect of Applied Voltage. Polymers.

[31] Luong-Van E., Grondahl L., Chua K.N., Leong K.W., Nurcombe V., Cool S.M. (2006). Controlled release of heparin from poly(epsilon-caprolactone) electrospun fibers. Biomaterials.

[32] Leonés A., Mujica-Garcia A., Arrieta M.P., Salaris V., Lopez D., Kenny J.M., Peponi L. (2020). Organic and Inorganic PCL-Based Electrospun Fibers. Polymers.

[33] Ye L., Wu X., Mu Q., Chen B., Duan Y., Geng X., Gu Y., Zhang A., Zhang J., Feng Z.-G. (2011). Heparin-Conjugated PCL Scaffolds Fabricated by Electrospinning and Loaded with Fibroblast Growth Factor 2. J. Biomater. Sci. Polym. Ed..

[34] Górecka Ż., Idaszek J., Kołbuk D., Choińska E., Chlanda A., Święszkowski W. (2020). The effect of diameter of fibre on formation of hydrogen bonds and mechanical properties of 3D-printed PCL. Mater. Sci. Eng. C.

[35] Han Y., Xu Y., Zhang S., Li T., Ramakrishna S., Liu Y. (2020). Progress of Improving Mechanical Strength of Electrospun Nanofibrous Membranes. Macromol. Mater. Eng..

[36] Yaseri R., Fadaie M., Mirzaei E., Samadian H., Ebrahiminezhad A. (2023). Surface modification of polycaprolactone nanofibers through hydrolysis and aminolysis: A comparative study on structural characteristics, mechanical properties, and cellular performance. Sci. Rep..

[37] Khunová V., Kováčová M., Olejniková P., Ondreáš F., Špitalský Z., Ghosal K., Berkeš D. (2022). Antibacterial Electrospun Polycaprolactone Nanofibers Reinforced by Halloysite Nanotubes for Tissue Engineering. Polymers.

[38] Suresh A.K., Doktycz M.J., Wang W., Moon J.W., Gu B., Meyer H.M., Hensley D.K., Allison D.P., Phelps T.J., Pelletier D.A. (2011). Monodispersed biocompatible silver sulfide nanoparticles: Facile extracellular biosynthesis using the γ-proteobacterium *Shewanella oneidensis*. Acta Biomater..

[39] Cialdai F., Risaliti C., Monici M. (2022). Role of fibroblasts in wound healing and tissue remodeling on Earth and in space. Front. Bioeng. Biotechnol..

[40] Farooq M., Khan A.W., Kim M.S., Choi S. (2021). The Role of Fibroblast Growth Factor (FGF) Signaling in Tissue Repair and Regeneration. Cells.

[41] Farahani M., Shafiee A. (2021). Wound Healing: From Passive to Smart Dressings. Adv. Heal. Mater..

[42] Ahmed M., Zayed M., El-Dek S., Hady M.A., El Sherbiny D.H., Uskoković V. (2021). Nanofibrous ε-polycaprolactone scaffolds containing Ag-doped magnetite nanoparticles: Physicochemical characterization and biological testing for wound dressing applications in vitro and in vivo. Bioact. Mater..

[43] Zivari-Ghader T., Hamishehkar H., Shokouhi B., Kosari-Nasab M., Farahpour M.R., Memar M.Y., Davaran S., Hanaee J., Rashidi M.-R., Mehrali M. (2024). Chitosan-Alginate Hydrogel Enriched with *Hypericum perforatum* Callus Extract for Improved Wound Healing and Scar Inhibition. ACS Appl. Mater. Interfaces.

[44] Poshina D., Sokolova N., Nono-Tagne S., Ahmadi-Nohadani H., Gofman I., Mishanin A., Golovkin A., Skorik Y., Otsuka I. (2024). Electrospinning of methacrylated alginate for tissue engineering applications. RSC Adv..

