# Supplementary material for: Multifunctional Biological Performance of Electrospun PCL Scaffolds Formulated with Silver Sulfide Nanoparticles
Source: Polymers (Basel). 2025 Jan 17;17(2):230. doi: 10.3390/polym17020230 (PMC11768394; doi:10.3390/polym17020230)
Supplement: Supplementary file 1 [file polymers-17-00230-s001.zip › polymers-3363778-supplementary.pdf]

Supplementary Materials: Multifunctional Biological Performance of Electrospun PCL Scaffolds Formulated with Silver Sulfide Nanoparticles

M.C. Torres-Pedroza [1], A. F. Martínez-Ávila [2], K. O. Juárez-Moreno [3], Miriam Estévez [3], L. Álvarez-Contreras [4], M. E. Cruz-Soto [5], L. Granados-López [6], Noé Arjona [1], and B. L. España-Sánchez [1,*]

1 Centro de Investigación y Desarrollo Tecnológico en Electroquímica SC, Parque Tecnológico Querétaro s/n Sanfandila, Pedro Escobedo, Querétaro, C.P. 76703, México; mtorres@cideteq.mx (M.C.T.-P.); wvelazquez@cideteq.mx (N.A.)
2 Facultad de Ciencias Naturales, Universidad Autónoma de Querétaro, Av. De las Ciencias s/n Juriquilla, Querétaro, C.P. 76230, México.; amartinez174@alumnos.uaq.mx
3 Centro de Física Aplicada y Tecnología Avanzada, Universidad Nacional Autónoma de México, Blvd. Juriquilla No. 3001, Querétaro, C.P. 76230, México.; kjuarez@fata.unam.mx (K.O.J.-M.), miries@fata.unam.mx (M.E.)
4 Centro de Investigación en Materiales Avanzados S.C. Complejo Industrial Chihuahua, Chihuahua, C.P. 31136, México; lorena.alvarez@cimav.edu.mx
5 Coordinación Nacional de Investigación en Ciencias de la Salud, Universidad del Valle de México Campus Querétaro , Blvd. Juriquilla 1000 A, Del. Santa Rosa Jáuregui, Querétaro, C.P. 76230, México; lorena.alvarez@cimav.edu.mx
6 Anatomía Patológica Querétaro, Hospital La Joya, Torre Momentum, Paseo de la República No. 13020, Querétaro, C.P. 76100, México. dralucerogranados@gmail.com
* Correspondence: lespana@cideteq.mx

Table S1. Comparative table of electrospinning parameters adjustment to obtain PCL/Hep/Ag2S nanostructured scaffolds.

Sample	Voltage (Kv)	Pump Flow (μL/h)	Distance (cm)	Collector Speed (rpm)	Fiber Morphology
PCL/Hep/Ag_2S NPs	10	6	10	1500	Twisted fibers
		6	15	1500	Irregular fibers
		12	10	1500	Irregular fibers
		12	15	1500	Fibers with beads
	12	6	15	1500	Irregular fibers
		12	15	1500	Twsited irregular fibers
	15	6	10	1500	Twisted fibers with beads
		6	15	1500	Fibers with beads
		12	10	1500	Regular nanofibers with beads
		12	15	1500	Homogeneous nanofibers
	18	12	15	1500	Irregular fibers

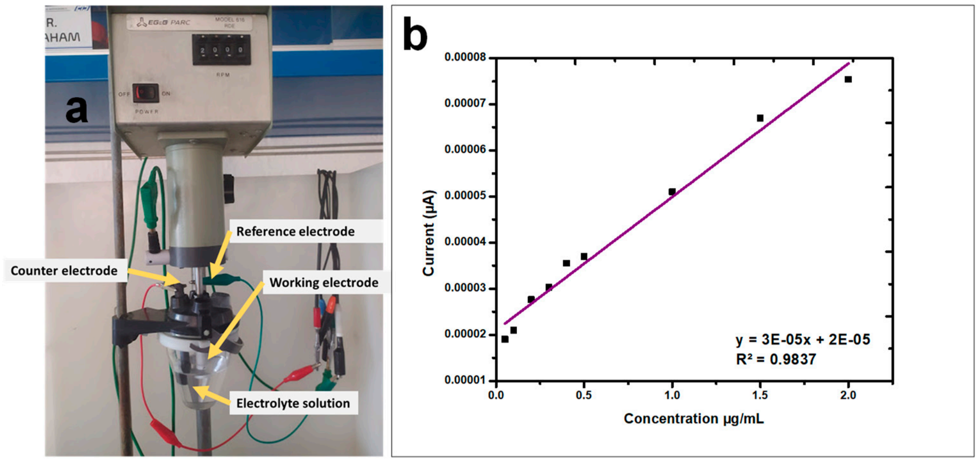

Figure S1. Ag ion release test by cyclic voltammetry: a) Three-electrode configuration and b) Calibration curve of Ag_2S NPs.

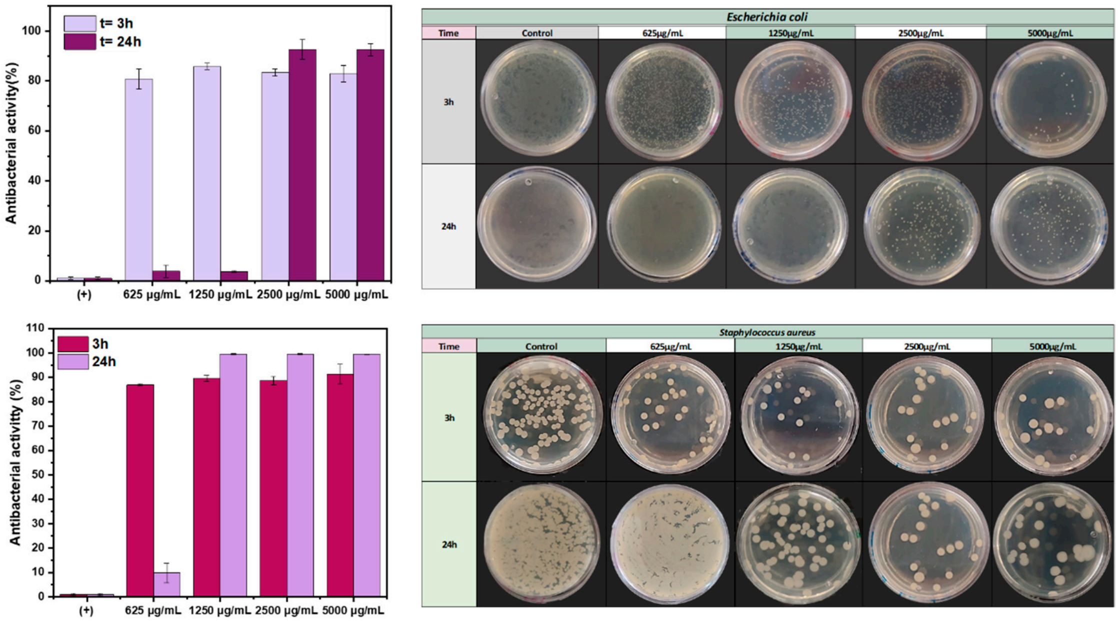

Figure S2. Kinetics of antibacterial activity (AA) of Ag_2S NPs after 3 and 24 h of contact against Gram (-) *E. coli* and *S. aureus*.

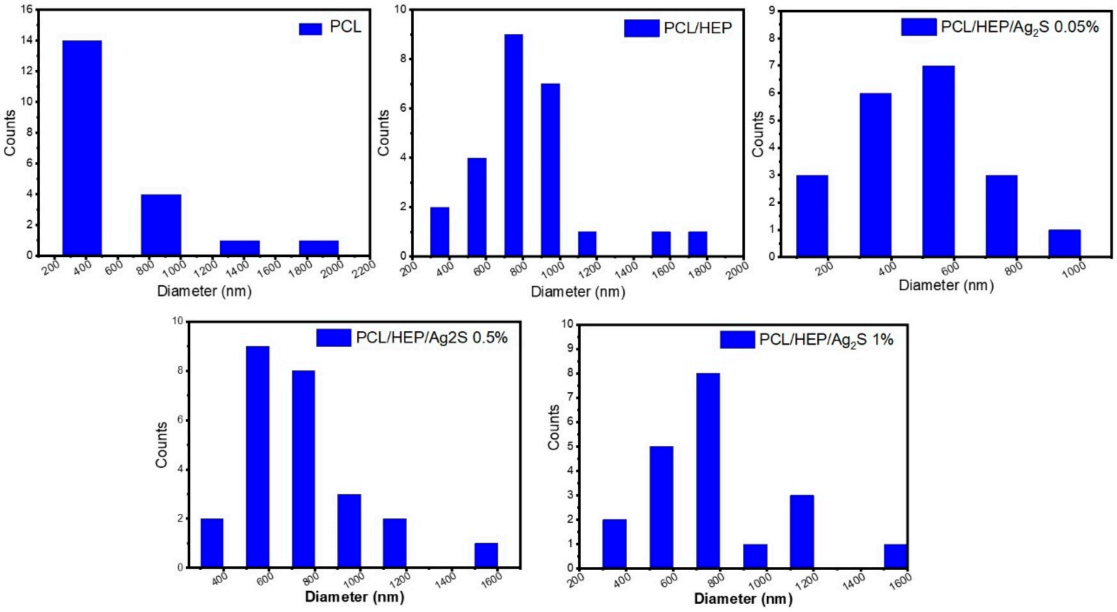

Figure S3. Fiber diameter distribution obtained from the electrospun of PCL, PCL/Hep, and the formulation of different amounts of Ag2S NPs (PCL/Hep/Ag_2S).

(a)

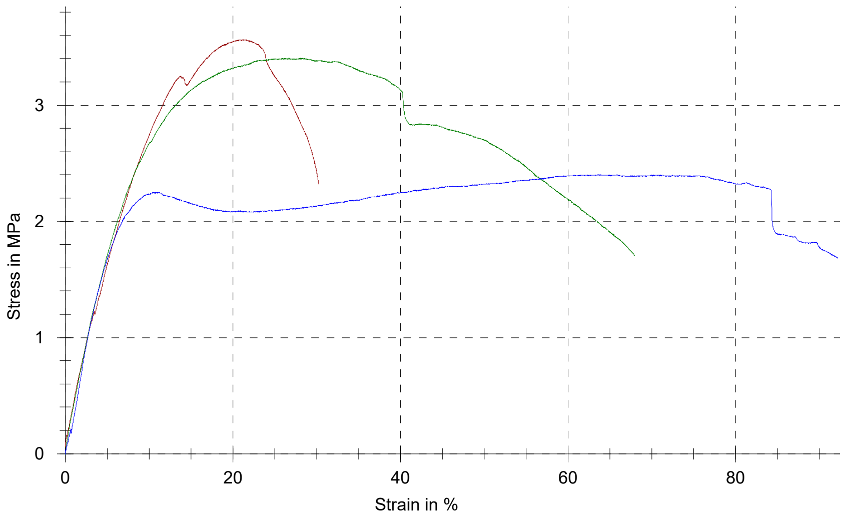

(b)

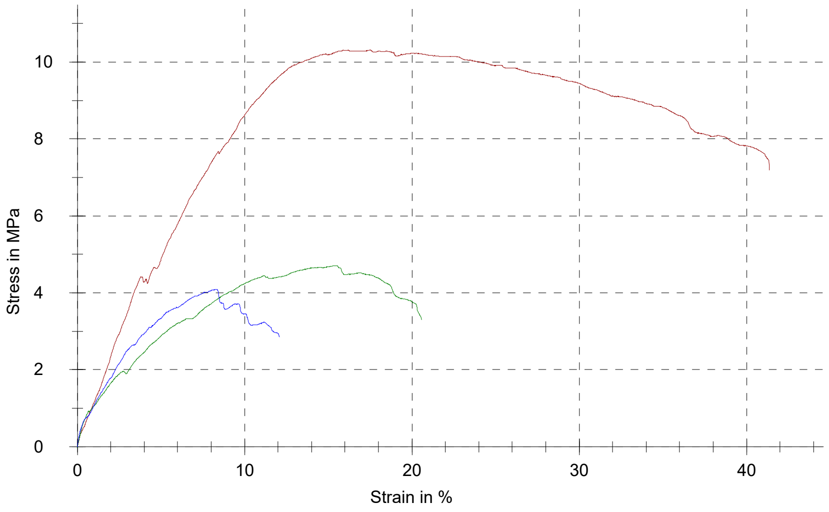

(c)

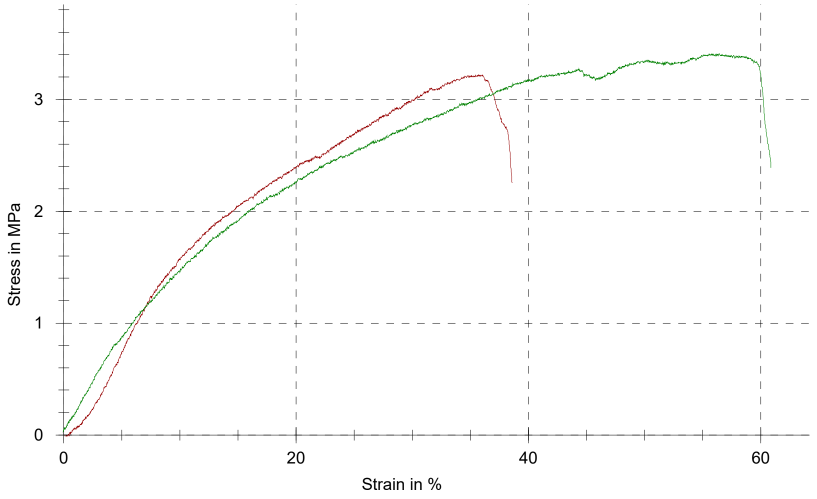

Figure S4. Stress-strain curves of (**a**) PCL, (**b**) PCL/HEP, and (**c**) PCL/HEP/Ag2S 0.5 %.
